# Brian Barraclough MD, FRCP, FRACP, FRCPsych, DPM

**DOI:** 10.1192/bjb.2025.10203

**Published:** 2026-06

**Authors:** Peter Tyrer

Formerly Senior Lecturer in Psychiatry, University of Southampton, UK

**Figure:**
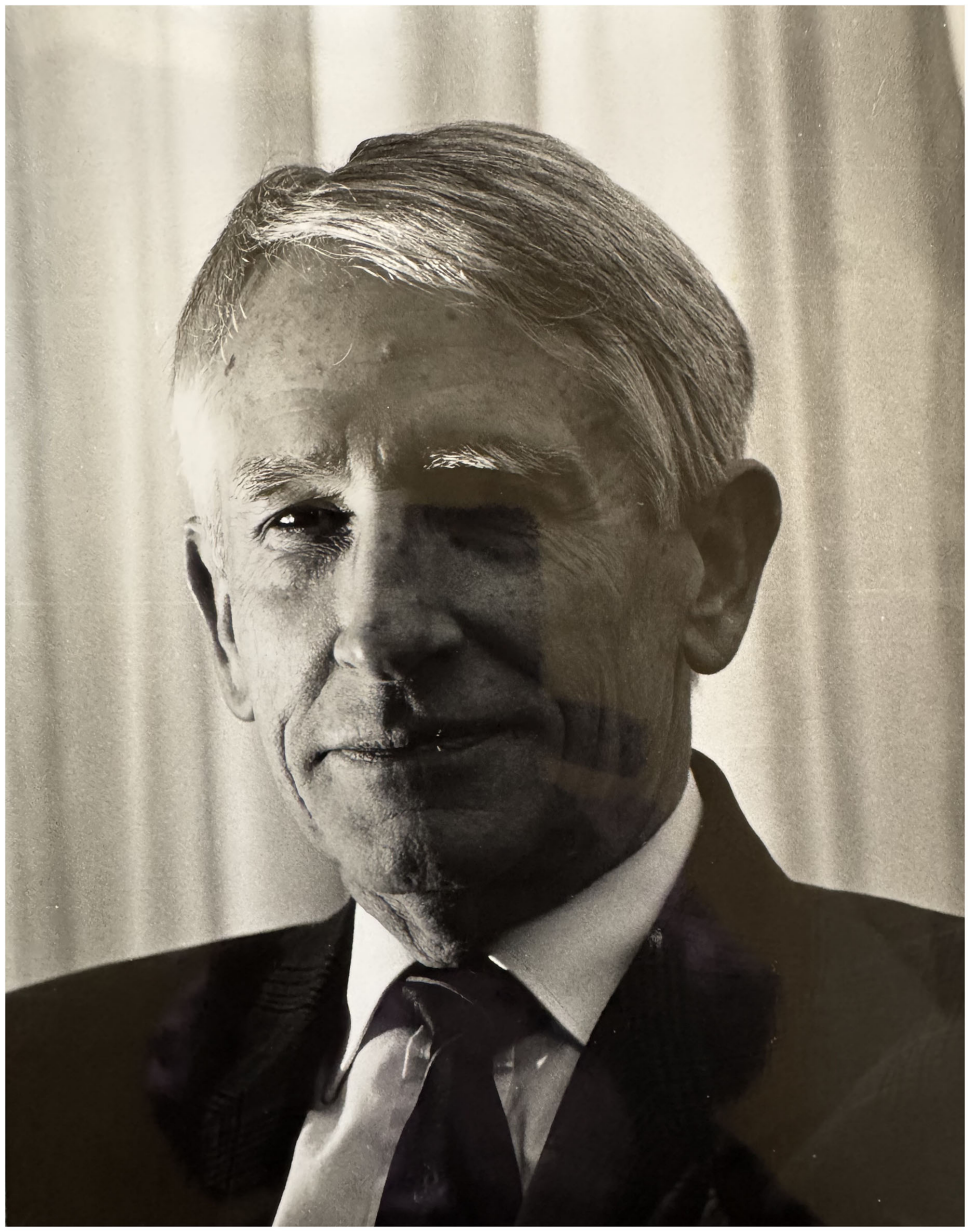

Between the 1970s and 1990s, Brian Barraclough’s research on clinical and social aspects of suicide contributed enormously to our understanding of the subject. His work continues to be cited in psychiatric literature and in policy documents.

Born on 16 June 1933, Brian was the elder of the two sons of Arthur Henry Barraclough, an Auckland businessman, and Dulcie Muriel, née Paterson. Both parents were grandchildren of mid-19th-century settlers to New Zealand or Australia. Links with English and Scots relatives who had not migrated had been lost and Brian felt entirely a New Zealander.

Brian was educated at Takapuna Primary and Takapuna Grammar Schools (TGS). At TGS he won Mr Short’s Shield as the best mathematician, the Horrock’s Cup for winning the half mile and was the senior tennis champion. Not having any idea what to do, other than get to university, he accepted his father’s suggestion to study chemistry. But a spell in hospital for pulmonary tuberculosis in 1951 attracted him to medicine and he graduated MB ChB from the University of Otago in 1957, after a mediocre undergraduate career. His choice of psychiatry resulted from the influence of Harold Bourne, a cultured Londoner, who became a lecturer in psychiatry at Otago in 1955 and made the subject a fascinating one to study.

In 1961, after 3 years in hospital posts in Christchurch, Brian passed the examination for the Membership of the Royal Australian College of Physicians (MRACP) and spent a year with Bourne at Dunedin Hospital, where he picked up the essentials of psychiatry. Then, in 1962, he travelled to London as a ship’s surgeon, to be interviewed for a place on the Maudsley Hospital’s training scheme. The Maudsley was then the world’s premier postgraduate training hospital for psychiatry and Brian felt very fortunate to be accepted. At the time, European psychiatry had not recovered from the war and US psychiatry was ruled by a sterile version of psychoanalysis.

A piece of original research was a requirement for the Academic Diploma in Psychological Medicine (DPM), the London University diploma exclusive to the Maudsley, and Brian found this more enjoyable than any other part of the course. In his investigation of appendicectomy, he found higher scores for symptoms of anxiety and depression in those with a normal appendix than in those with true appendicitis.

After a bare pass in the DPM examination he joined the Medical Research Council’s Clinical Psychiatry Research Unit at Chichester. Sir Aubrey Lewis, the doyen of academic psychiatry at the Maudsley Hospital, had recommended him to the director, Peter Sainsbury, who introduced him to the subject of suicide, one that became a key part of his research life.

Peter Sainsbury gave Brian the task of studying the health and social aspects of suicide by interviewing surviving relatives, a novel approach at the time. The published finding of a high level of mental illness among suicides^1^ became a standard reference. Further studies on suicide continued, including an examination, with Daphne Shepherd, of the aftermath for widows and children.

When the Royal College of Psychiatrists was founded in 1971, Brian became, through an electoral quirk, a member of Council, the Executive and Finance Committee and, from 1973 to 1976, chairman of the College Research Committee. He was a member of the Journal Committee from 1970 to 1980 and for the *British Journal of Psychiatry* deputy editor to Edward Hare from 1973 to 1976. He also initiated the series ‘In conversation with’, interviewing well-known psychiatric personalities and publishing these in the *Bulletin of the Royal College of Psychiatrists* as a regular journal feature.

In 1980, with Sainsbury’s retirement and the closure of the Chichester Unit imminent, Brian moved to the University of Southampton as senior lecturer in psychiatry under Professor James Gibbons, and as honorary consultant to the Royal South Hants Hospital. Clinical work on an acute admission ward came as a culture shock after the rarefied environment of full-time research.

Until his retirement on health grounds in 1994, Brian taught undergraduates and postgraduates, treated patients, took a part in National Health Service administration and, most enjoyable of all, supervised 4th-year undergraduate research projects. Research was a feature of the Southampton MB course and required virtual full-time student commitment for a year.

After retiring, Brian returned to research as an honorary senior lecturer under Gibbons’ successor, Professor Chris Thompson. With Clare Harris he published two highly cited papers on suicide as an outcome of medical and mental disorders^2,3^ and with Martin Brown on suicide pacts. An interest in historical aspects of suicide produced an examination of the Bible suicides. He also supervised five doctoral students.

In 2000, Brian returned to New Zealand to live in Devonport, Auckland, near where he had grown up. He was appointed an honorary clinical associate professor at Auckland University. He turned to medical history, editing the unpublished autobiography of DW Carmalt Jones, an Oxford graduate who had been professor of medicine at Otago University from 1919 to 1939, and writing the first volume of an autobiography, *A Partly Anglicised Kiwi: A Psychiatrist Remembers*,^4^ reviewed in this journal (2021), with a second volume due for publication shortly.

Brian was married three times: to Patricia Garrett MA from 1957 to 1962, to Maureen Blasdale from 1963 to 1982 and to Jennifer Hughes MD FRCP from 1983. With Maureen he had two children, Geoffrey and Helen. They live in London and in Frome, Somerset. He also leaves three grandchildren.

Brian Barraclough saw his professional life as a succession of pieces of unexpected good luck: to be born in New Zealand, to get tuberculosis, recover and move into medicine, to meet Harold Bourne and be shown an interesting medical specialty, to pass the MRACP, to join the Maudsley Hospital and receive a liberal education there, mainly in conversation with his better educated colleagues, to be taken on by Peter Sainsbury who taught him the art of scientific research and improved his manners, to be recruited by James Gibbons to Southampton University and to return to beautiful New Zealand with an adequate pension and a very happy marriage.
